# Supplementary material for: Brain Natriuretic Peptide Protects Cardiomyocytes from Apoptosis and Stimulates Their Cell Cycle Re-Entry in Mouse Infarcted Hearts
Source: Cells. 2022 Dec 20;12(1):7. doi: 10.3390/cells12010007 (PMC9818267; doi:10.3390/cells12010007)
Supplement: Supplementary file 1 [file cells-12-00007-s001.zip › Supplementary Figure S1.docx]

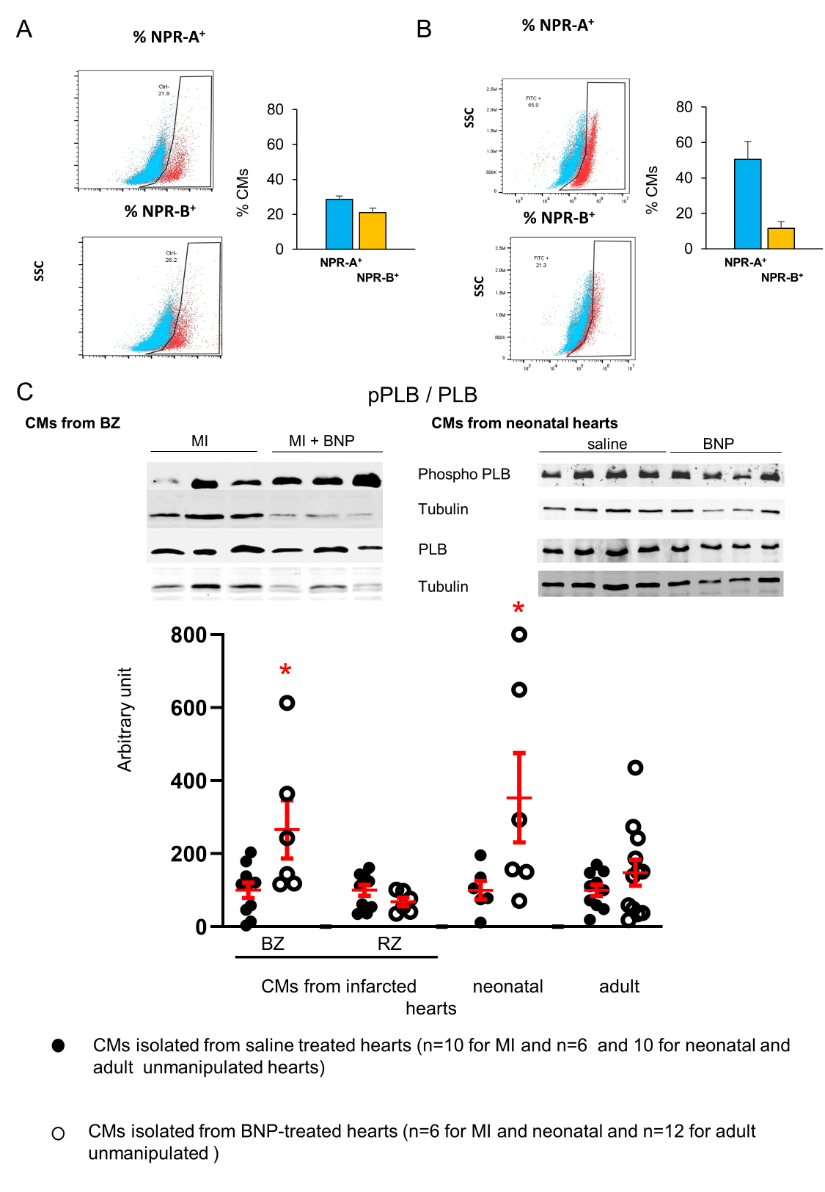


**Supplemental Figure 1. Neonatal and adult cardiomyocytes express NPR-A and NPR-B receptors and are able to respond directly to BNP stimulations. A-B.** NPR-A or NPR-B expression assessed by flow cytometry analysis on isolated neonatal (**A;** n=6 different isolations) and adult (**B;** n=4 different isolations) cardiomyocytes. Results represented as means ± SEM **C.** pPLB/PLB ratio in CMs isolated from adult infarcted, unmanipulated neonatal or adult hearts treated *in vivo* with saline (black circles) or BNP (white circles). Blots stained with antibodies against phospho phospholamban (pPLB), phospholamban (PBL) and Tubulin (used as loading control). Results of BNP treated CMs expressed relatively to the average of saline treated CMs*.* Individual isolations represented and the means ± SEM represented in red ***** p<0.05 versus CMs isolated from saline treated hearts.
