# Supplementary material for: Brain Natriuretic Peptide Protects Cardiomyocytes from Apoptosis and Stimulates Their Cell Cycle Re-Entry in Mouse Infarcted Hearts
Source: Cells. 2022 Dec 20;12(1):7. doi: 10.3390/cells12010007 (PMC9818267; doi:10.3390/cells12010007)
Supplement: Supplementary file 1 [file cells-12-00007-s001.zip › Supplementary Figure S2.docx]

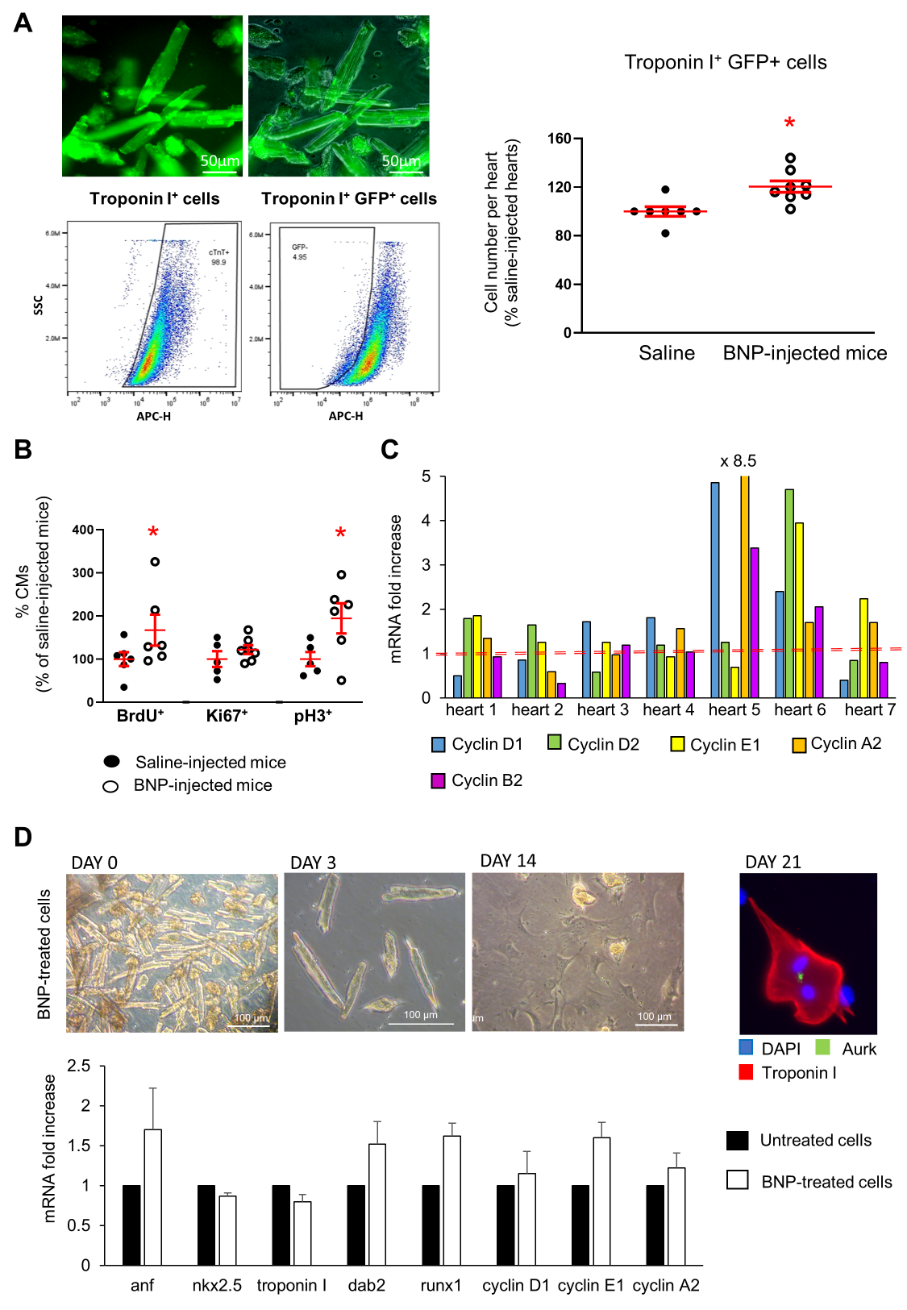


CMs analyzed by qRT-PCR after 7 days of culture for the expressions of mRNAs coding for the atrial natriuretic peptide (anf), nkx2.5, dab2 and runx1, both re-expressed during CM dedifferentiation, and the different cyclins (D1, E1 and A2). n=3 different cell cultures. Results of BNP-treated CMs related to those of untreated cells. Data are means ±SEM. * p< 0.05 versus untreated cells.

**Supplementary Figure 2. BNP treatment stimulates cardiomyocytes in unmanipulated adult hearts to re-enter in the cell cycle**. **A.** CMs isolated from unmanipulated Myh6 MerCreMer mice, treated 2 weeks with BNP or saline and injected with Tamoxifen 2 weeks before BNP injections. Flow cytometry analysis performed on these isolated CMs with an antibody against Troponin I. CMs identified as Troponin I^+^ GFP^+^ cells, counted**. B**. Immunostainings using antibodies against α actinin and BrdU or Ki67 or pH3 performed in adult unmanipulated hearts from saline or BNP-injected mice. The percentages of CMs expressing these proliferative markers measured on at least 10 pictures per mouse. **A-B:** Individual values represented and the means ± SEM represented in red. n=6 saline and BNP-injected hearts. **C**. mRNA expression coding for cyclin D1, D2, E1, A2 and B2. Results of CMs isolated from adult BNP-treated hearts expressed as fold increase above the levels in CMs isolated from saline-treated hearts (represented by the red dotted line, n= 8 saline-injected adult hearts). 7 different CM cell isolations from BNP-treated hearts represented**. D**. Adult cardiomyocytes isolated from 6-week-old unmanipulated C57BL/6 hearts cultured *in vitro* with or without BNP (10 nM). Representative pictures showing the evolution of the cell culture and the presence of Aurkb^+^ Troponin I^+^ cells in BNP-treated cells.
