# Supplementary material for: Brain Natriuretic Peptide Protects Cardiomyocytes from Apoptosis and Stimulates Their Cell Cycle Re-Entry in Mouse Infarcted Hearts
Source: Cells. 2022 Dec 20;12(1):7. doi: 10.3390/cells12010007 (PMC9818267; doi:10.3390/cells12010007)
Supplement: Supplementary file 1 [file cells-12-00007-s001.zip › Supplementary Figure S3.docx]

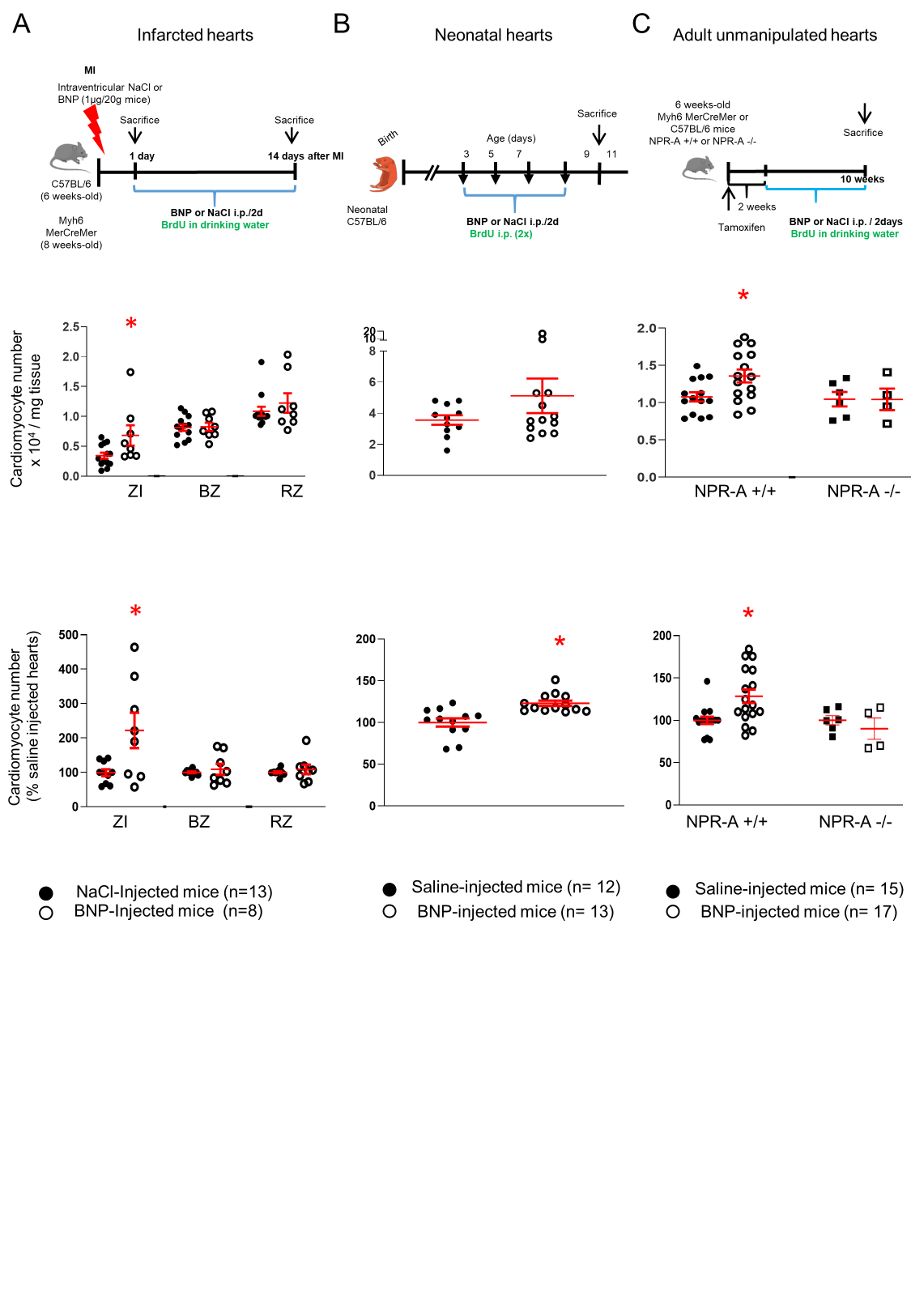


**Supplemental Figure 3. BNP injections in infarcted adult mice (A), in neonatal (B) and adult (C) unmanipulated mice lead to increased number of cardiomyocytes.** Cardiomyocytes isolated from BNP or saline injected heart counted and the number related to the tissue weight or to the number found in saline-injected mice. Individual isolation represented and the means ± SEM in red ***** p<0.05 versus saline treated hearts.
