# Supplementary material for: Brain Natriuretic Peptide Protects Cardiomyocytes from Apoptosis and Stimulates Their Cell Cycle Re-Entry in Mouse Infarcted Hearts
Source: Cells. 2022 Dec 20;12(1):7. doi: 10.3390/cells12010007 (PMC9818267; doi:10.3390/cells12010007)

**Supplemental Figure 4. BNP treatment increases the number of neonatal cardiomyocytes *in vitro*. A.** Representative pictures of 2-weeks-old neonatal CM cell culture treated with different BNP concentrations (0-1000 nM). **B**. CM cell number related to the number of untreated CMs of the same isolation. n= 6 different cell cultures. For each experiment, the number of Troponin I^+^ cells / 0.9 mm^2^ counted on at least 10 different pictures. **C**. Cardiomyocyte cross-sectional area measured in BNP treated cells and related to the area of untreated CMs. At least 60 CMs were measured per condition and cell culture (n= 6). **D**. Percentages of mononucleated CMs in untreated and BNP-treated conditions. At least 2700 CMs originating from 5 different cell cultures were evaluated in each condition. **E-F**. Detections by immunostainings of CMs (Troponin I^+^ cells) expressing Ki67, pH3 or Aurora B (Aurkb) proliferative markers in untreated or BNP-treated (10-100 nM) cultures. **G.** Percentages of CMs expressing these markers in BNP-treated cells related to untreated cells (n=6-8 different cell cultures). **H.** mRNA expression coding for cyclin genes D1, E1, A2 and B2. Results of BNP-treated CMs related to those of untreated cells.


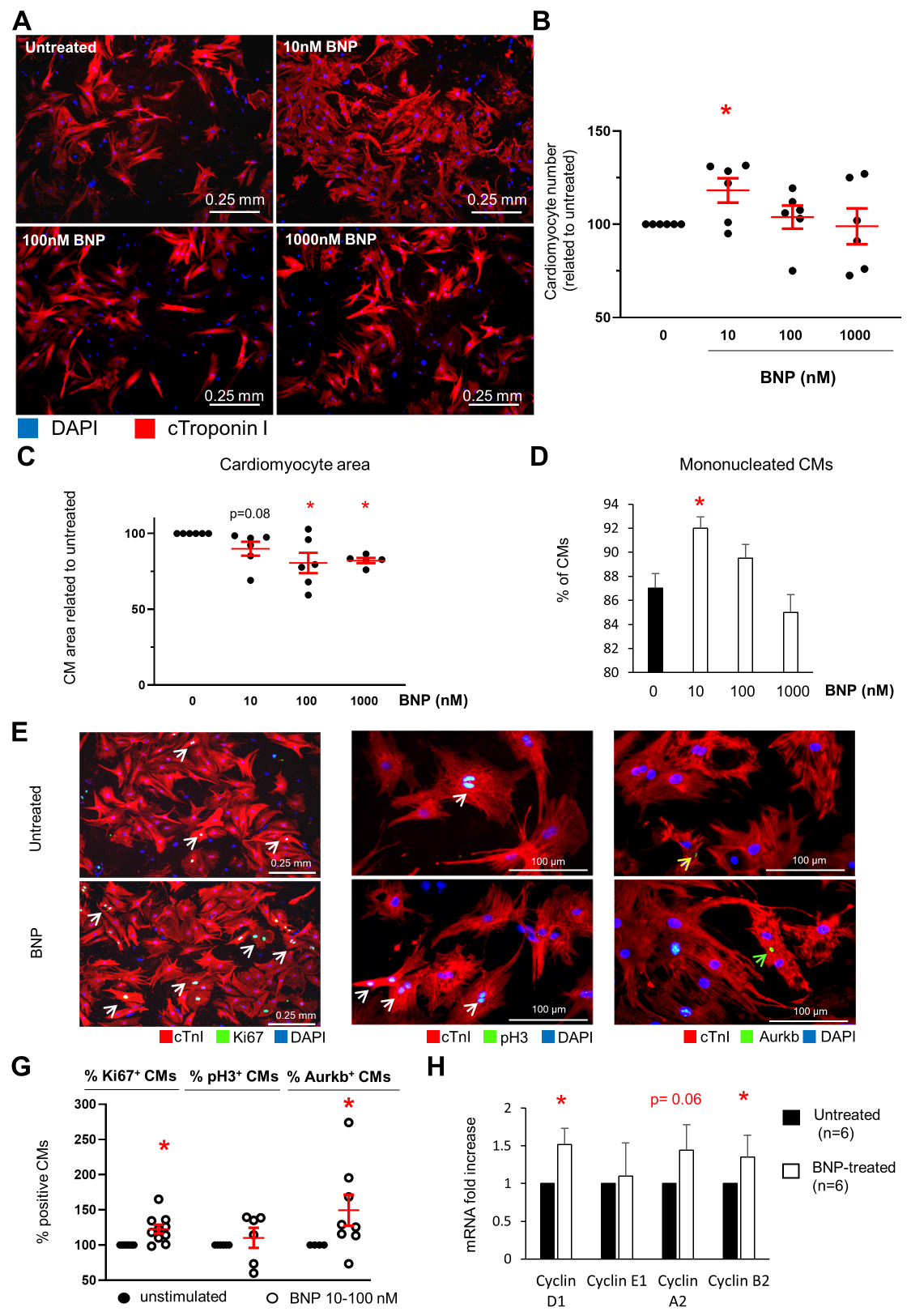

Supplement: Supplementary file 1 [file cells-12-00007-s001.zip › Supplementary Figure S4.docx]
