# Supplementary material for: Brain Natriuretic Peptide Protects Cardiomyocytes from Apoptosis and Stimulates Their Cell Cycle Re-Entry in Mouse Infarcted Hearts
Source: Cells. 2022 Dec 20;12(1):7. doi: 10.3390/cells12010007 (PMC9818267; doi:10.3390/cells12010007)
Supplement: Supplementary file 1 [file cells-12-00007-s001.zip › Supplementary Figure S5.docx]

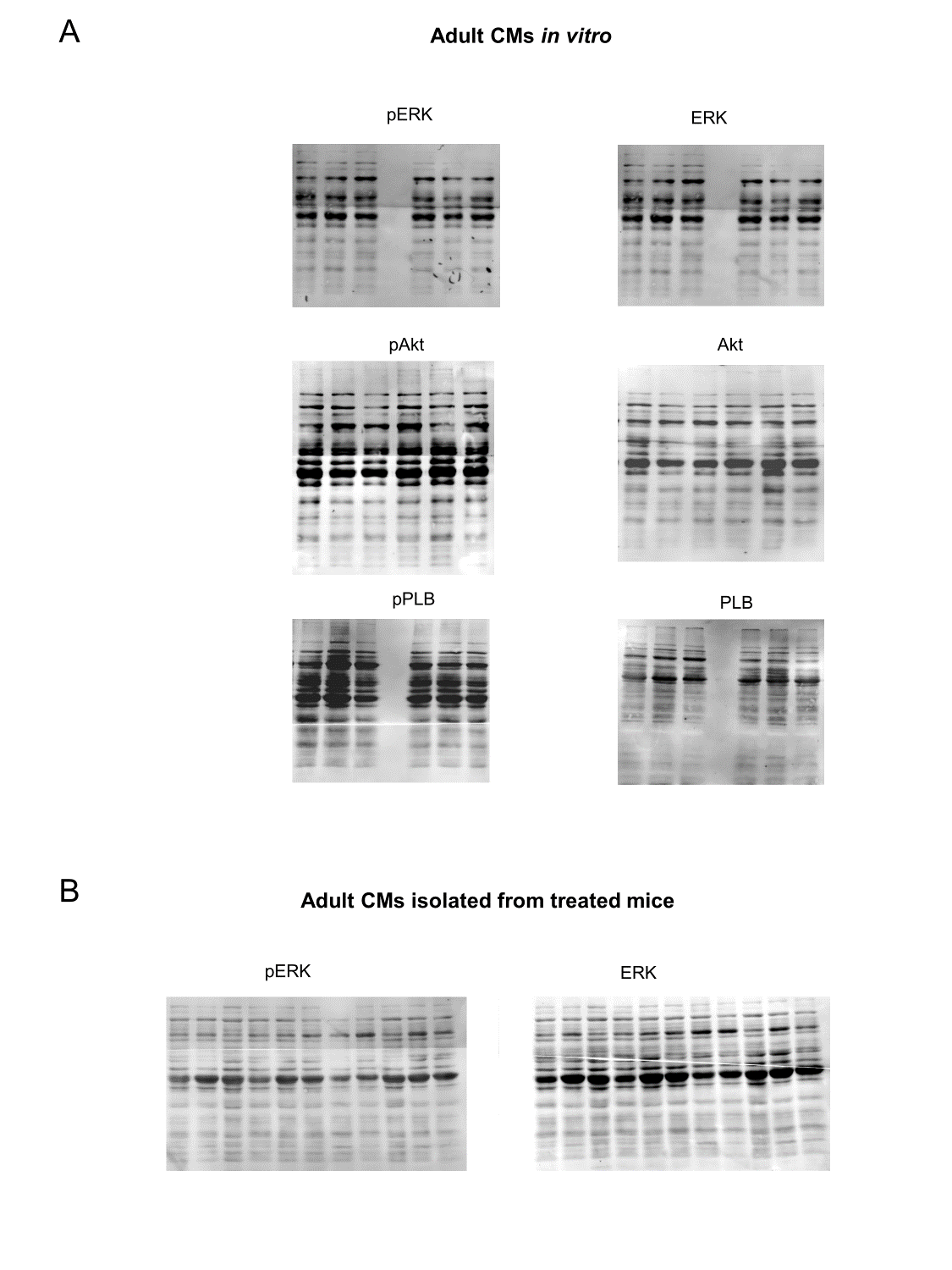


**Supplemental Figure 5.** Examples of total protein stainings of membranes used to measure pERK, ERK, pAkt and Akt levels in adult CMs stimulated *in vitro* with BNP (**A**) or in adult CMs isolated from treated mice (**B**). The results of the Figure 6 A and B are related to these total protein levels.
