# Supplementary material for: Brain Natriuretic Peptide Protects Cardiomyocytes from Apoptosis and Stimulates Their Cell Cycle Re-Entry in Mouse Infarcted Hearts
Source: Cells. 2022 Dec 20;12(1):7. doi: 10.3390/cells12010007 (PMC9818267; doi:10.3390/cells12010007)
Supplement: Supplementary file 1 [file cells-12-00007-s001.zip › Supplementary File S1.docx]

**Supplementary File 1:** Antibodies used in flow cytometry analysis, immunohistology and western blot analysis.

|  | **Species** | **Dilution** | **Reference Technics** | |  |
| --- | --- | --- | --- | --- | --- |
| **Primary antibodies**  α-actinin | mouse | 1/50 | Sigma A7811 | Immunohistology | |
| Aurkb | rabbit | 1/1000 | Abcam ab139188 | Immunohistology | |
| BrdU | rat | 1/100 | Abcam ab6326 | Immunohistology | |
| cleaved caspase 3 | rabbit | 1/400 | Cell Signaling 9661 | Immunohistology | |
| GFP | rabbit | 1/1000 | Abcam ab290 | Immunohistology | |
| Phospho Histone H3 (S10) | rabbit | 1/100 | Millipore 06-570 | Immunohistology | |
| Ki67 | rat | 1/1000 | ebioscience 14-5698-80 | Immunohistology | |
| Laminin | rabbit | 1/200 | Sigma L9393 | Immunohistology | |
| NPR-A | rabbit | 1/50 | Abcam ab70848 | Immunohistology | |
| NPR-B | rabbit | 1/100 | Abcam ab139188 | Immunohistology | |
| Troponin I | goat | 1/100 | Santa Cruz Biotechnology SC-8118 | Immunohistology | |
| Akt | rabbit | 1/1000 | Cell Signaling | Western Blot | |
| Phospho-Akt | rabbit | 1/500 | Cell Signaling | Western Blot | |
| Bax | rabbit | 1/1000 | Cell Signaling | Western Blot | |
| Bcl-2 | rabbit | 1/1000 | Cell Signaling | Western Blot | |
| cleaved caspase 8 | rabbit | 1/1000 | Cell Signaling | Western Blot | |
| cleaved caspase 3 | rabbit | 1/1000 | Cell Signaling | Western Blot | |
| Erk | rabbit | 1/3000 | Cell Signaling | Western Blot | |
| NPR-B | goat | 1/20 | Santa Cruz SC-34421 | Flow cytometry | |
| NPR-A | rabbit | 1/50 | Abcam ab70848 | Flow cytometry | |
| NPR-C  Phospho-Erk | mouse  rabbit | 1/100  1/2000 | GeneTex  Cell Signaling | Flow cytometry  Western Blot | |
| p38 | rabbit | 1/1000 | Cell Signaling | Western Blot | |
| Phospho-P38 | rabbit | 1/500 | Cell Signaling | Western Blot | |
| phospholamban | mouse | 1/1000 | Abcam | Western Blot | |
| Phospho-phospholamban | rabbit | 1/500 | Millipore | Western Blot | |
| Troponin I | goat | 1/50 | Abcam ab56357 | Flow cytometry | |
| Tubulin | mouse | 1/10000 | Sigma T5168 | Western Blot | |
| **Secondary antibodies** |  |  |  |  | |
| Anti-rabbit Alexa 488 | donkey | 1/1000 | Molecular Probes A21206 | Immunohistology | |
| Anti-rabbit Alexa 594 | donkey | 1/1000 | Molecular Probes A21207 | immunohistology | |
| Anti-goat Alexa 594 | donkey | 1/1000 | Molecular Probes A11058 | Immunohistology | |
| Anti-mouse Alexa 647 | goat | 1/1000 | Molecular Probes A21240 | Immunohistology | |
| Anti-rat 647 | donkey | 1/500 | Jackson Immuno 712-605-150 | Immunohistology | |
| Anti-rat Alexa 488 | donkey | 1/1000 | Molecular Probes A21208 | Immunohistology | |
| Anti-rat biotinylated | goat | 1/200 | Vector BA-9400 | Immunohistology | |
| Steptavidine Alexa 594 |  | 1/1000 | Molecular Probes S11227 | Immunohistology | |
| Anti-rabbit Alexa 680 | goat | 1/5000 | Molecular Probes A21109 | Western Blot | |
| Anti-mouse IRDye 800 | goat | 1/10000 | Rockland Immuno-chemicals 610-132-121 | Western Blot | |
| Anti-goat APC-conjugated  Anti-mouse IgG2b FITC | chicken  goat | 1/10  1/1000 | R&D systems F0108  Molecular Probe A21141 | Flow cytometry  Flow cytometry | |
