# Supplementary material for: Brain Natriuretic Peptide Protects Cardiomyocytes from Apoptosis and Stimulates Their Cell Cycle Re-Entry in Mouse Infarcted Hearts
Source: Cells. 2022 Dec 20;12(1):7. doi: 10.3390/cells12010007 (PMC9818267; doi:10.3390/cells12010007)
Supplement: Supplementary file 1 [file cells-12-00007-s001.zip › Supplementary File S2.docx]

**Supplementary File 2:** List of primers used in qRT-PCR.

| **Gene** | **Forward primer** | **Reverse primer** | **Product size (bp)** |
| --- | --- | --- | --- |
| **anf** | ACAGGATTGGAGCCCAGAGC | GTCCATGGTGCTGAAGTTTATTC | 337 |
| **acta1** | TGGACTTCGAGAATGAGATGG | TCGTCCTGAGGAGAGAGAGC | 509 |
| **cyclin A2** | ATGTCAACCCCGAAAAACTG | GCAGTGACATGCTCATCGTT | 157 |
| **cyclin B2** | AGCTCCCAAGGATCGTCCTC | TGTCCTCGTTATCTATGTCCTCG | 116 |
| **cyclin E1** | GAAAGAAGAAGGTGGCTCCGAC | GTTAGGGGTGGGGATGAAAGAG | 190 |
| **cyclin D1** | TGAGAACAAGCAGACCATCC | TGAACTTCACATCTGTGGCA | 71 |
| **cyclin D2** | GGATGATGAAGTGAACACACTCAC | GGATCTTCCACAGACTTGGATCC | 180 |
| **dab2** | TGCTCGTGATGTGACAGACA | AGGGTCATTAGGGCCTCACT | 225 |
| **hif1α** | CTGTCATCTCACTATGGGCA | CCAAGTCCGAGCAGGAATTT | 259 |
| **myh6** | AACCAGAGTTTGAGTGACAGAATG | ACTCCGTGCGGATGTCAA | 130 |
| **myh7** | ATGAGACGGTGGTGGGTTT | CTTTCTTTGCCTTGCCTTTG | 117 |
| **nkx2.5** | CAAGTGCTCTCCTGCTTTCC | GTCCAGCTCCACTGCCTTCT | 130 |
| **npr1** | CCAATTATGGCTCCCTGCT | CGGTACAAGCTCCCACAAAT | 198 |
| **npr2** | TCATGACAGCCCATGGGAAA | GGTGACAATGCAGATGTTGG | 209 |
| **npr3** | GGCTCAATGAGGAGGATTACGTG | AATCTTCCCGCAGCTCTCGATG | 555 |
| **runx1** | GATGGCACTCTGGTCACCG | GCCGCTCGGAAAAGGACA | 298 |
| **troponin T** | GCGGAAGAGTGGGAAGAGACA | CCACAGCTCCTTGGCCTTCT | 127 |
| **18S** | ACTTTTGGGGCCTTCGTGTC | GCCCAGAGACTCATTTCTTCTTG | 96 |
